# Risk of Spina Bifida and Maternal Cigarette, Alcohol, and Coffee Use during the First Month of Pregnancy

**DOI:** 10.3390/ijerph10083263

**Published:** 2013-08-02

**Authors:** Corey M. Benedum, Mahsa M. Yazdy, Allen A. Mitchell, Martha M. Werler

**Affiliations:** Slone Epidemiology Center at Boston University, 1010 Commonwealth Ave. Boston, MA 02215, USA; E-Mails: cbenedum@bu.edu (C.M.B.); mahsa@bu.edu (M.M.Y.); allenmit@bu.edu (A.A.M.)

**Keywords:** spina bifida, alcohol consumption, caffeine consumption, cigarettes, folic acid, birth defects

## Abstract

This study was conducted to assess the association between the risks of spina bifida (SB) in relation to cigarette, alcohol, and caffeine consumption by women during the first month of pregnancy. Between 1988–2012, this multi-center case-control study interviewed mothers of 776 SB cases and 8,756 controls about pregnancy events and exposures. We evaluated cigarette smoking, frequency of alcohol drinking, and caffeine intake during the first lunar month of pregnancy in relation to SB risk. Logistic regression models were used to calculate adjusted odds ratios and 95% confidence intervals. Levels of cigarette smoking (1–9 and ≥10/day), alcohol intake (average ≥4 drinks/day) and caffeine intake (<1, 1, and ≥2 cups/day) were not likely to be associated with increased risk of SB. Further, results were similar among women who ingested less than the recommended amount of folic acid (400 μg/day).

## 1. Introduction

Spina bifida (SB), a neural tube defect (NTD), is a serious birth defect affecting approximately 35 per 100,000 live births [1]. SB, like other NTDs, occurs when the neural tube fails to properly close within the first 28 days after conception [2]. Maternal folic acid (FA) ingestion during the periconceptional period has been found to decrease the risk of giving birth to an infant with a NTD [3,4,5,6]. Based upon these findings, mandatory FA fortification of enriched cereal grains began in 1998 in the U.S. and Canada [7]. After U.S. fortification, an increase in the blood folate levels of adults was observed [8,9], but not all women of childbearing age ingest the recommended amount of 400 μg per day [7].

Because SB continues to occur among mothers who have ingested at least 400 μg per day of FA, other factors have been considered to explain the etiology of this birth defect. Among these, and based on their established or hypothesized teratogenic properties in humans, are cigarette smoking, alcohol drinking, and caffeine consumption [10,11,12,13,14]. Despite the positive association between maternal smoking and some birth defects, as well as the presence of known teratogenic chemicals in cigarettes [11], the association between maternal smoking and NTDs has been inconclusive, with study findings ranging from a protective effect to an increase in risk [10,11,14,15]. Extreme alcohol intake is a known human teratogen, causing Fetal Alcohol Syndrome in some exposed fetuses. NTDs are not a typical component of Fetal Alcohol Syndrome, but case reports suggest a possible link [16]. Epidemiological studies of moderate or low alcohol intakes and NTDs are inconclusive [11,14,17]. Caffeine intake in late gestation affects fetal cardiovascular function, but its effects in early gestation on organogenesis in general and neural tube development in particular are not known [18]. One epidemiologic study showed a slight increase in SB risk for all sources of caffeine [12], whereas three others showed no association [13,17,18].

FA metabolism is believed to be altered by exposure to cigarette smoke, alcohol, and caffeine. Smokers have lower plasma folate levels after adjustment for folate intake [19,20]. Alcohol interferes with folate transport and metabolism [21,22]. Lower plasma folate concentrations were observed in coffee drinkers in a cross-sectional study, but dietary folate or supplemental folic acid were not taken into account [23].

Utilizing data collected in the Boston University Slone Epidemiology Center Birth Defects Study, we tested the hypotheses that the risk of SB is associated with smoking, alcohol drinking, and coffee consumption during the first 28 days after the last menstrual period (LMP). Additionally, we investigated whether the risk would be greater in women who failed to ingest the recommended amount of folic acid.

## 2. Methods

### 2.1. Study Population

The Slone Epidemiology Center Birth Defects Study is an on-going case-control study in North America, which began in 1976 and has been described in detail elsewhere [24,25,26,27]. Cases of birth defects were identified through birth hospitals, tertiary care centers, and birth defect registries in Massachusetts (1976+); Philadelphia, PA (1976+); Toronto, ON, Canada (1976–2005); San Diego County, CA (2001+); and parts of New York State (2004+). Beginning in 1990, therapeutic abortions after 12 weeks’ gestation and fetal deaths occurring after 20 weeks’ gestation were eligible for the study if identified; however, these pregnancies were not routinely ascertained. Beginning in 1993, the study began ascertaining non-malformed controls from the same birth population that gave rise to cases. For the years prior to 1993, infants born with major malformations other than the one under study or those born with only minor malformations (e.g., hip clicks, toe anomalies) or nonstructural defects (e.g., cystic fibrosis) were used as controls.

The present study was restricted to subjects interviewed between 1988 and 2012 when questions on changes in behaviors were asked. Cases of SB were excluded if they had a conjoined twin, chromosomal anomaly, Mendelian-inherited disorder, a known syndrome, amniotic bands, or a body wall defect. Cases were then reviewed by a clinical geneticist to ensure that they met the case definition. For 1988–1992, controls were infants with minor malformations or non-structural defects; for 1993–2012, control subjects were non-malformed infants.

Maternal interviews were conducted within six months of delivery by trained study nurses; interviews were conducted in person until 1998 and, thereafter by telephone. The interview consisted of questions pertaining to socio-demographic factors, reproductive history, illness during pregnancy, details on prescription and over-the-counter medication use (including vitamins), and behavioral risk factors (e.g., cigarette smoking, alcohol drinking, and coffee consumption). Cases and controls whose maternal exposure information was missing were excluded from the specific analysis. To assess dietary intake, the long version Willett Food Frequency Questionnaire (FFQ) was administered from 1988–1997; it was replaced with a modified, shortened, version in 1998.

### 2.2. Smoking

For the period beginning two months before and throughout pregnancy, mothers were asked the average number of cigarettes smoked per day before and after any changes in amount and the dates of those changes. Using the maternal-reported average and accounting for changes, we calculated an estimated average number of cigarettes smoked by determining the total number of cigarettes smoked during the 28 days after the LMP and then averaging the total number over the 28 days. Mothers were categorized according to the calculated average amount of cigarettes smoked per day (<1, 1–9, and ≥10) during the first lunar month after the LMP.

### 2.3. Alcohol

Mothers were asked about the average number of drinking days per week (frequency) and the average number of drinks per drinking day (intensity) of alcohol consumption two months prior to and during pregnancy, including changes in patterns of intake and the date of any such change. Maternal-reported averages and the change dates were used to estimate the total number of drinks per day and drinking days for the 28 days. A calculated average of the 28-day period was then determined. Mothers were categorized as a heavy drinker if they reported ≥4 drinks per day at any time during the first lunar month of pregnancy. Mothers were also classified, using the calculated average, according to their frequency of consumption in days per week (<1, 1, 2, and ≥3) and the intensity of consumption as the number of drinks per drinking day (<1, 1, 2, and ≥3) during the first lunar month after the LMP. 

### 2.4. Coffee

From 1998 through 2012, mothers were asked about the average number of cups of caffeinated coffee, tea, and soda consumed two months before and during their pregnancy; changes in frequency and the timing of any changes were also recorded. Before 1998, data on changes in frequency use were not collected. Changes in the interview occurring in 2005 led to different categorizations for tea and soda that were incompatible with previous years and therefore the main analysis was restricted to coffee intake from 1998 through 2012. To assess the influence of other sources of caffeine, a sensitivity analysis was performed using coffee, soda, and tea data from 1998 through 2005. For both analyses, mothers were categorized by the average number of caffeinated beverages/coffee consumed per day (none, <1, 1, and ≥2) during the first lunar month of pregnancy based on the total number over the 28 day period divided by 28 days, taking any changes into account.

### 2.5. Smoking, Alcohol, and Coffee Interaction

We then assessed potential interactions among the exposure variables. The interactions were examined by pairs (smoking/heavy drinking, smoking/coffee, heavy drinking/coffee) and all together (smoking/heavy drinking/coffee). Women who were in the highest exposure category for each variable were then compared to women who were in the reference category.

### 2.6. Folic Acid Intake

FA intake during the first lunar month of pregnancy was calculated by summing the average daily folic acid intake from supplements and fortified foods. Natural folate was also included, but discounted by 30% due to its lower bioavailability compared to synthetic folic acid [28]. The residual energy adjustment method [29] was used to adjust all dietary variables for total caloric intake. Participants were then categorized according to total folic acid intake (<400 and ≥400 μg/day). Participants with extreme caloric intakes (<500 or >4,000 kcal/day) or incomplete FFQ (≥3 missing items) were excluded from analyses involving folic acid intake, with two exceptions: Women who reported ≥400 μg per day of folic acid from vitamin supplements or who reported not taking any supplements containing folic acid were retained in the analysis. The former group was retained because those taking folic acid-containing vitamins would fall into the ≥ 400 μg per day stratum regardless of dietary intake. The latter group was retained based upon previous studies in which non-users would not likely reach the ≥ 400 μg per day stratum from diet alone [7]; therefore they were placed in the <400 μg per day category.

### 2.7. Statistical Analysis

Multiple logistic regression models were used to calculate crude (cORs) and adjusted odds ratios (aORs) and 95% confidence intervals (CIs) for each level of cigarette, alcohol and coffee exposure, with the non-use of each as the reference category. Sociodemographic factors that were considered as potential confounders included: maternal race/ethnicity (non-Hispanic White, non-Hispanic Black, Hispanic, other), maternal age (<20, 20–24, 25–29, 30–34, ≥35 years), maternal education (<12, 12, >12 years), study center (Boston, MA, USA; Philadelphia, PA, USA; Toronto, ON, Canada; San Diego county, CA, USA; New York State, USA), body mass index (underweight, normal, overweight, obese; available for 1993 onward), non-steroidal anti-inflammatory (NSAID) drug use (yes, no), use of medication that is to known be a folic acid antagonist (yes, no), and amount of FA intake (<400 μg/day, ≥400 μg/day). Variables that changed estimates by more than 10% were kept in the final model. A sub-analysis was performed for each exposure, in which we excluded women who had been diagnosed with diabetes prior to the index pregnancy. Data were stratified by study year (1988–1997 and 1998–2012) due to interview differences and by FA intake to assess for potential effect measure modification. All analyses were performed using SAS 9.2 software [30].

## 3. Results

A total of 511 cases of SB and 868 controls (135 controls with minor malformations) were included for the years 1988 through 1997, while 265 cases of SB and 7,888 non-malformed controls were included for the years after 1998. Distributions of sociodemographic factors are presented in Table 1. For the years 1988 through 1997, of those mothers who were eligible and were asked to participate, mothers of 80% of cases and 68% of controls agreed to be interviewed [31]. For the years 1998 through 2012, the equivalent proportions were 71% of cases and 67% of controls [32].

### 3.1. Smoking

Due to missing smoking information, six cases and five controls were excluded from the 1988–1997 smoking analysis and three controls were excluded from the 1998–2012 smoking analysis. For all years, cigarette smoking was more common among younger mothers and those with fewer years of education (Table 2). In the earlier years, smoking was more prevalent in cases (32%) compared to controls (22%), while in the later years little difference was seen between cases (18%) and controls (15%). Among the factors examined as possible confounders, study center, maternal education, NSAID use, and folic acid antagonist medication use, were found to meet the criterion for confounding (*i.e.*, addition of the variable caused more than a 10% change in the estimate) for the years 1988 through 1997 only maternal race/ethnicity and education met the criterion for confounding in the later years.

**Figure 1:**
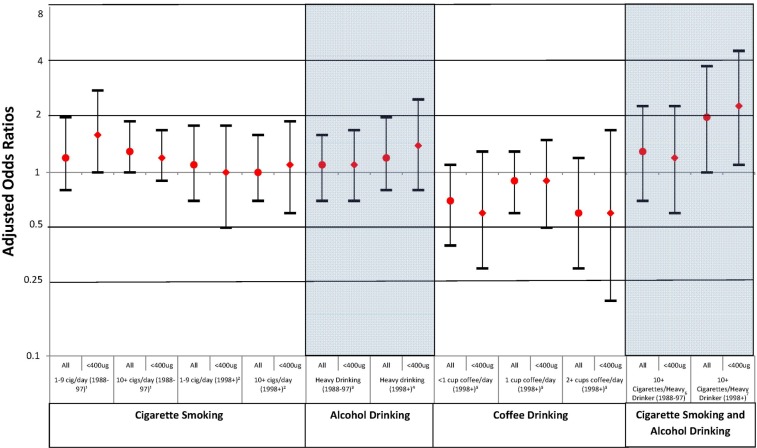
Adjusted Odds Ratios for Associations between Spina Bifida and Exposure for All Subjects and Subjects with <400μg Daily Folic Acid intake, Birth Defects Study 1988–2012.

^1^ Adjusted for maternal education, study center, NSAID use, and folic acid antagonist medication use; ^2^ Adjusted for maternal education and race/ethnicity; ^3^ Adjusted for maternal education; ^4^ Adjusted for NSAID use and folic acid antagonist medication use; ^5^ Adjusted for study center; ^6^ Adjusted for maternal education, study center, NSAID use, and folic acid antagonist medication use; ^7^ Adjusted for NSAID use and folic acid antagonist medication use.

For the years 1988 through 1997, no increased risk was observed for moderate smoking but there was a suggestion of an increased risk for heavier smokers (1–9 cigarettes/day: aOR: 1.2, 95% CI: 0.8, 2.0; ≥10 cigarettes/day: aOR: 1.3, 95% CI: 0.9, 1.7) (Table 3). No increased risks were found in the later years (1–9 cigarettes/day: aOR: 1.1, 95% CI: 0.7, 1.8 and ≥10 cigarettes/day: aOR: 1.0, 95% CI: 0.7, 1.6). When the data were stratified by folic acid intake, there was no appreciable change in risk among women with low FA intake (Figure 1). Furthermore, when women with pregestational diabetes were excluded from the smoking analysis there was no observable change in risks (1988–1993: 1–9 cigarettes/day: aOR: 1.3, 95% CI: 0.8, 2.0; ≥10 cigarettes/day: aOR: 1.3, 95% CI: 0.9, 1.7; 1998–2012: 1–9 cigarettes/day: aOR: 1.1, 95% CI: 0.7, 1.7; ≥10 cigarettes/day: aOR: 1.0, 95% CI: 0.6, 1.6) (data not shown). 

**Table 1:** Maternal Demographics of Spina Bifida Cases and Controls, Birth Defects Study, 1988–2012.

	1988–1997				1998–2012			
	Controls		SB Cases		Controls		SB Cases	
	n	%	N	%	n	%	n	%
**Total**	868		511		7,888		265	
*Maternal race/ethnicity*								
White, non-Hispanic	782	90.1	455	89.0	5,645	71.6	166	62.6
Black, non-Hispanic	46	5.3	30	5.9	662	8.4	28	10.6
Hispanic	17	2.0	16	3.1	896	11.4	50	18.9
Other ǂ	23	2.6	10	2.0	672	8.5	21	7.9
Missing	0	0.0	0	0.0	13	0.2	0	0.0
*Maternal age at conception*								
<20 years	24	2.8	44	8.6	555	7.0	20	7.6
20–24 years	91	10.5	102	20.0	1,149	14.6	43	16.2
25–29 years	298	34.3	164	32.1	2,065	26.2	88	33.2
30–34 years	325	37.4	144	28.2	2,641	33.5	72	27.2
≥35 years	130	15.0	57	11.2	1,457	18.5	42	15.9
Missing	0	0.0	0	0.0	21	0.3	0	0.0
*Maternal Education*								
< 12 years	55	6.3	83	16.2	720	9.1	40	15.1
12 years	187	21.5	181	35.4	1,452	18.4	64	24.2
> 12 years	625	72.0	247	48.3	5,708	72.4	160	60.4
Missing	1	0.1	0	0.0	8	0.1	1	0.4
*Study center (years in study)*								
Boston, MA (1976+)	314	36.2	195	38.2	3,991	50.6	41	15.5
Philadelphia (1976+)	186	21.4	155	30.3	1,567	19.9	91	34.3
Toronto (1979–2005)	368	42.4	161	31.5	645	8.2	60	22.6
San Diego (2001+)	0	0.0	0	0.0	1,131	14.3	38	14.3
New York (2004+)	0	0.0	0	0.0	554	7.0	35	13.2
BMI * (1993+)								
Underweight	60	6.9	11	2.2	376	4.8	7	2.6
Normal	449	51.7	78	15.3	4,814	61.0	128	48.3
Overweight	152	17.5	34	6.7	1,549	19.6	55	20.8
Obese	67	7.7	27	5.3	965	12.2	60	22.6
Missing	140	16.1	361	70.6	184	2.3	15	5.7
*Folic Acid*								
<400μg	561	64.6	386	75.5	3,499	44.4	123	46.4
≥400μg	297	34.2	112	21.9	4,222	53.5	131	49.4
Missing	10	1.2	13	2.5	167	2.1	11	4.2

ǂ Other include mixed non-Hispanic, Native Hawaiian, Pacific Islander, Asian, Native American, or other * BMI: Body Mass Index defined as 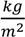

**Table 2:** Maternal Demographics According to Control Subject’s Exposure During the First Month of Pregnancy, Birth Defects Study, 1988–2012.

	Smoking (1988–1997)		Smoking (1998–2012)		Alcohol Consumption (1988–1997)		Alcohol Consumption (1998–2012)		Coffee Consumption (1998–2012)	
	<1	≥1	<1	≥1	No	Yes	<1	≥1	<1	≥1
	n (%)	n (%)	n (%)	n (%)	n (%)	n (%)	n (%)	n (%)	n (%)	n (%)
*Maternal race/ethnicity*										
White, non-Hispanic	606 (89.8)	176 (91.2)	4,721 (70.6)	924 (76.9)	354 (87)	428 (92.8)	2,769 (61.8)	2,876 (84.4)	3,777 (68.4)	1,868 (79)
Black, non-Hispanic	38 (5.6)	8 (4.1)	554 (8.3)	108 (9)	26 (6.4)	20 (4.3)	487 (10.9)	175 (5.1)	579 (10.5)	170 (7.2)
Hispanic	12 (1.8)	5 (2.6)	789 (11.8)	107 (8.9)	13 (3.2)	4 (0.9)	706 (15.8)	190 (5.6)	654 (11.8)	83 (3.5)
Otherǂ	19 (2.8)	4 (2.1)	610 (9.1)	62 (5.2)	14 (3.4)	9 (2)	509 (11.4)	163 (4.8)	502 (9.1)	242 (10.2)
Missing	0 (0)	0 (0)	12 (0.2)	1 (0.1)	0 (0)	0 (0)	9 (0.2)	4 (0.1)	11 (0.2)	2 (0.1)
*Maternal Age*										
<20 years	10 (1.5)	14 (7.3)	393 (5.9)	162 (13.5)	14 (3.4)	10 (2.2)	435 (9.7)	120 (3.5)	473 (8.6)	82 (3.5)
20–24 years	57 (8.4)	34 (17.6)	822 (12.3)	327 (27.2)	42 (10.3)	49 (10.6)	734 (16.4)	415 (12.2)	884 (16)	265 (11.2)
25–29 years	228 (33.8)	70 (36.3)	1,751 (26.2)	314 (26.1)	152 (37.3)	146 (31.7)	1,202 (26.8)	863 (25.3)	1,473 (26.7)	592 (25)
30–34 years	270 (40)	55 (28.5)	2,391 (35.8)	250 (20.8)	142 (34.9)	183 (39.7)	1,342 (30)	1,299 (38.1)	1,757 (31.8)	884 (37.4)
≥35 years	110 (16.3)	20 (10.4)	1,308 (19.6)	149 (12.4)	57 (14)	73 (15.8)	753 (16.8)	704 (20.7)	918 (16.6)	539 (22.8)
Missing	0 (0)	0 (0)	21 (0.3)	0 (0)	0 (0)	0 (0)	14 (0.3)	7 (0.2)	18 (0.3)	3 (0.1)
*Maternal Education*										
< 12 years	29 (4.3)	26 (13.5)	498 (7.4)	222 (18.5)	31 (7.6)	24 (5.2)	584 (13)	136 (4)	564 (10.2)	156 (6.6)
12 years	118 (17.5)	69 (35.8)	1,010 (15.1)	442 (36.8)	103 (25.3)	84 (18.2)	971 (21.7)	481 (14.1)	1,002 (18.1)	450 (19)
> 12 years	527 (78.1)	98 (50.8)	5,170 (77.3)	538 (44.8)	273 (67.1)	352 (76.4)	2,918 (65.1)	2,790 (81.9)	3,950 (71.5)	1,758 (74.3)
Missing	1 (0.1)	0 (0)	8 (0.1)	0 (0)	0 (0)	1 (0.2)	7 (0.2)	1 (0)	7 (0.1)	1 (0)
*Folic Acid*										
<400	419 (62.1)	142 (73.6)	2,705 (40.5)	794 (66.1)	251 (61.7)	310 (67.2)	2,055 (45.9)	1,444 (42.4)	2,459 (44.5)	1,040 (44)
400+	249 (36.9)	48 (24.9)	3,848 (57.6)	374 (31.1)	151 (37.1)	146 (31.7)	2,300 (51.3)	1,922 (56.4)	2,945 (53.3)	1,277 (54)
Missing	7 (1)	3 (1.6)	133 (2)	34 (2.8)	5 (1.2)	5 (1.1)	125 (2.8)	42 (1.2)	119 (2.2)	48 (2)
*Study center (years in study)*										
Boston, MA (1976+)	243 (36)	71 (36.8)	3,324 (49.7)	667 (55.5)	133 (32.7)	181 (39.3)	2,182 (48.7)	1,809 (53.1)	2,619 (47.4)	1,372 (58)
Philadelphia (1976+)	153 (22.7)	33 (17.1)	1,311 (19.6)	256 (21.3)	120 (29.5)	66 (14.3)	942 (21)	625 (18.3)	1,201 (21.7)	366 (15.5)
Toronto (1979-2005)	279 (41.3)	89 (46.1)	536 (8)	109 (9.1)	154 (37.8)	214 (46.4)	334 (7.5)	311 (9.1)	435 (7.9)	210 (8.9)
San Diego (2001+)	0 (0)	0 (0)	1,050 (15.7)	81 (6.7)	0 (0)	0 (0)	674 (15)	457 (13.4)	880 (15.9)	251 (10.6)
New York (2004+)	0 (0)	0 (0)	465 (7)	89 (7.4)	0 (0)	0 (0)	348 (7.8)	206 (6)	388 (7)	166 (7)

ǂ Other include mixed non-Hispanic, Native Hawaiian, Pacific Islander, Asian, Native American, or other

### 3.2. Alcohol

Heavy drinking was examined by comparing mothers who drank ≥4 drinks per drinking day any time during the first month to mothers who drank less or none (Table 3). Maternal education was the only factor that met the confounding criterion in the earlier years while NSAID use and folic acid antagonist medication use were identified for the later years. Slightly more mothers of cases reported heavy alcohol consumption than mothers of controls, but the difference was confounded by maternal education in the early years and NSAID and folic acid antagonist medication in later years, resulting in ORs that approximated the null. There was no change in the risk after stratification by folic acid intake for women who had low intake levels (Figure 1). When women with pregestational diabetes were excluded, there was no change in aORs (1988–1993: aOR: 1.1, 95% CI: 0.7, 1.7; 1998–2012: aOR: 1.2, 95% CI: 0.8, 2.0) (data not shown).

One case from the early period and seven controls from the later period were excluded from the alcohol analysis on frequency and intensity due to missing data on alcohol intake. Sociodemographic factors meeting the criterion for confounding were study center, maternal education, and FA intake. For frequency and intensity of alcohol intake, the reference groups were defined as mothers reporting <1 drinking day per week and <1 drink per drinking day, respectively. When both frequency and intensity were considered together, women in both of these lower levels of intake constituted the reference group. Considered separately, neither frequency nor intensity measures was associated with increased SB risk, but women who were both frequent (≥3 days/week) and intense (≥3 drinks/day) drinkers were twice as prevalent among SB cases as controls (Table 4). However, the approximate 2-fold crude odds (cOR = 2.1) of a frequent and intense drinking mother giving birth to an SB baby compared to the referent was confounded by FA intake, study center by year, and maternal education (aOR = 1.2). Additionally, the exclusion of women with pregestational diabetes did not result in any observable changes in aORs (data not shown).

### 3.3. Coffee

Caffeine intake from coffee consumption was analyzed for data between 1998 through 2012; no subjects were excluded from the coffee analysis due to missing data. Only study center met the criterion for confounding. Compared to mothers who reported consuming no coffee, no increase in risk was observed for daily coffee drinkers (<1 cup/day aOR: 0.7, 95% CI: 0.4, 1.1; 1 cup/day aOR: 0.9, 95% CI: 0.6, 1.3; ≥2 cups/day aOR: 0.6, 95% CI: 0.3, 1.2). Furthermore, there was no observed change in risk among mothers who had low intake levels of folic acid (Figure 1). When other sources of caffeine were included for the sensitivity analysis, no elevated risks were identified (<1 cup/day aOR: 0.8, 95% CI: 0.5, 1.4; 1 cup/day aOR: 0.9, 95% CI: 0.4, 1.6; ≥2 cups/day: aOR: 1.0, 95% CI: 0.6, 1.7). Exclusion of women with pregestational diabetes did not result in any observable changes in aORs (<1 cup/day aOR: 0.7, 95% CI: 0.5, 1.4; 1 cup/day aOR: 0.9, 95% CI: 0.6, 1.3; ≥2 cups/day: aOR: 0.6, 95% CI: 0.3, 1.2) (data not shown).

**Table 3:** Association between Spina Bifida and Alcohol, Smoking, and Caffeine During the First Month of Pregnancy, Birth Defects Study, 1988–2012.

	Cases		Controls		cOR (95% CI)	aOR (95% CI)
	n	%	n	%		
**Smoking**						
*1988–1997^ 1^*						
None	342	66.9	675	77.8	Ref	Ref
1–9 cig/day	38	7.4	53	6.1	1.4 (0.9, 2.2)	1.2 (0.8, 2.0)
10+ cigs/day	125	24.5	135	15.6	1.8 (1.4, 2.4)	1.3 (0.9, 1.7)
Missing	6	1.2	5	0.6	-	-
*1998+ ^2^*						
None	218	82.3	6,686	84.8	Ref	Ref
1–9 cig/day	22	8.3	522	6.6	1.3 (0.8, 2.0)	1.1 (0.7, 1.8)
10+ cigs/day	25	9.4	677	8.6	1.1 (0.7, 1.7)	1.0 (0.7, 1.6)
Missing	0		3	<0.1	-	-
**Alcohol**						
*1988–1997^ 3^*						
No heavy drinking	456	89.2	805	92.7	Ref	Ref
Heavy drinking ^φ^	55	10.8	63	7.3	1.5 (1.1, 2.3)	1.1 (0.7, 1.6)
*1998+^ 4^*						
No heavy drinking	239	90.6	7,385	93.6	Ref	Ref
Heavy drinking ^φ^	25	9.4	496	6.3	1.6 (1.0, 2.4)	1.2 (0.8, 2.0)
Missing	1	0.4	7	<0.1	-	-
**Smoking and Alcohol use**						
*1988–1997^ 5^*						
No smoking/ not heavy drinking	322	69.0	646	79.8	Ref	Ref
10+ cigs per day/ heavy drinking	29	6.2	24	3.0	2.4 (1.4, 4.2)	1.3 (0.7, 2.3)
Missing	6	1.3	5	0.6	-	-
*1998+ ^6^*						
No smoking/ not heavy drinking	214	88.1	6,433	87.4	Ref	Ref
10+ cigs per day/ heavy drinking	11	4.5	149	2.0	2.2 (1.2, 4.2)	2.0 (1.0, 3.8)
Missing	1	0.4	10	0.1	-	-
**Caffeine from coffee**						
*1998+^ 7^*						
None	205	77.4	5,523	70.0	Ref	Ref
<1 cup/day	21	7.9	844	10.7	0.7 (0.4, 1.1)	0.7 (0.4, 1.1)
1 cup/day	31	11.7	1084	13.7	0.8 (0.5, 1.1)	0.9 (0.6, 1.3)
2+ cups/day	8	3.0	437	5.5	0.5 (0.2, 1.0)	0.6 (0.3, 1.2)

^φ^ Heavy drinking: Maternal report of drinking an average 4+ drinks per sitting at any time during the first lunar month; ^1^ Adjusted for maternal education, study center, NSAID use, and folic acid antagonist medication use; ^2^ Adjusted for maternal education and race/ethnicity; ^3^ Adjusted for maternal education; ^4^ Adjusted for NSAID use and folic acid antagonist medication use; ^5^ Adjusted for maternal education, study center, NSAID use, and folic acid antagonist medication use; ^6^ Adjusted for maternal education, race/ethnicity, NSAID use, and folic acid antagonist medication use; ^7^ Adjusted for study center.

**Table 4:** Association between Spina Bifida and Alcohol Consumption by Frequency and Intensity During the First Month of Pregnancy, Birth Defects Study, 1988–2012.

			Average number of drinking days per week				Total
			<1	1	2	3+	
Average number of drinks per drinking day	**<1**	Cases	424	0	0	0	424
		Controls	4,882	3	0	1	4,886
		cOR	**Ref**	-	-	-	**Ref**
		aOR (95% CI) *	-	-	-	-	-
	**1**	Cases	76	15	9	14	114
		Controls	487	239	178	270	1,174
		cOR	1.8	0.7	0.6	0.6	1.1
		aOR (95% CI) *	0.7 (0.5, 1.1)	0.5 (0.3, 1.1)	0.5 (0.2, 1.1)	0.6 (0.3, 1.1)	0.7 (0.5, 0.9)
	**2**	Cases	61	20	21	11	113
		Controls	615	383	297	198	1,493
		cOR	1.1	0.6	0.8	0.6	0.9
		aOR (95% CI) *	1 (0.7, 1.5)	0.6 (0.4, 1.1)	0.8 (0.5, 1.4)	0.6 (0.3, 1.2)	0.8 (0.6, 1.1)
	**3+**	Cases	52	36	15	22	125
		Controls	513	316	249	123	1,201
		cOR	1.2	1.3	0.7	2.1	1.2
		aOR (95% CI) *	0.7 (0.5, 1.0)	1 (0.6, 1.6)	0.3 (0.2, 0.6)	1.2 (0.6, 2.3)	0.7 (0.6, 1.0)
**Total**		Cases	613	71	45	47	
		Controls	6,497	941	724	592	
		cOR	**Ref**	0.9	0.7	0.9	
		aOR (95% CI) *	-	0.8 (0.5, 1.1)	0.8 (0.4, 0.8)	0.7 (0.5, 1.3)	

* Adjusted for folic acid intake, study center by year, and maternal education.

### 3.4. Smoking, Alcohol, and Coffee Interaction

Because there was only one case in both the highest exposure group of caffeine and smoking, and none in the highest caffeine and heavy drinking, we did not assess these interactions in relation to SB risk. Women who reported both smoking 10+ cigarettes per day and heavy alcohol drinking were compared to women who did not smoke and were not heavy drinkers. Confounders identified in the early years were study center, maternal education, NSAID use, and folate antagonist medication use. In the later years confounders that met the criterion were maternal education, race/ethnicity, NSAID use, and folate antagonist medication use. Prior to 1998, there was no observed association for SB risk and the combination of 10+ cigarettes and heavy drinking (aOR: 1.3 95% CI: 0.7, 2.3). However, in the later years, a modest association was observed but the confidence interval included the null (aOR: 2.0 95% CI: 1.0, 3.8). After stratifying women according to folic acid intake, there was little change in risk among mothers in both periods; however the elevated risk among mothers in the later years was more apparent (aOR: 2.3 95% CI: 1.1, 4.6) (Figure 1).

## 4. Discussion

In this case-control study the risk for SB does not appear to be associated with cigarette smoking, alcohol consumption, and coffee consumption at the levels of intake observed in the study. These null results, with narrow CIs, are consistent with many previous reports [12,13,14,17,33,34,35,36,37,38,39,40,41,42] but are in contrast to a smaller number of studies with positive associations [11,12,38,43].

### 4.1. Smoking

Our results for the earlier years of the study showed a small increase in risk for SB at each level of cigarette smoking, with a slight dose effect. In the later years, however, both levels of smoking exposure resulted in a null association with SB risk. The discontinuity between findings in the two time periods could be due to several factors. First, beginning in 1998, interviews were conducted over the phone as opposed to in-person. If in-person interviews elicit more accurate responses, then our findings from the more recent years would be more vulnerable to bias. A second possible explanation is the use of different controls prior to and following 1993; controls with minor malformations and certain medical conditions were used prior to 1993. However, a post hoc analysis of data collected before and after that change in the composition of control subjects revealed similar results (data not shown). Another possibility is uncontrolled confounding by factors that vary in distribution between the two time periods. For example, if FA is a true confounder and we tended to underestimate intakes, a larger proportion of women in the early years would be affected by such misclassification when intakes in general were lower, as observed in other population-based studies of intakes and blood folate levels [9]. Thus, uncontrolled confounding by FA intake would be greater in the earlier years. Finally, the percentage of heavier smokers has decreased over the past two decades in our study and in the general population of pregnant women [44,45,46]. If cigarette smoking has a threshold effect on SB risk, our null findings in the later years might be due the lower levels of smoking within the highest exposure category. The finding of no association has been observed in other studies [14,42,43]. One study broadly categorized smoking exposure as ≤5 and >5 cigarettes per day, while two others used finer categories of exposure (1–19, ≥20 and 1–14, 15–24, ≥25 cigarettes/day).

### 4.2. Alcohol

In the present study, neither frequent nor intense alcohol consumption was associated with SB risk, which is consistent with previous studies [11,17,34]. While the frequency of mothers who drank at least three drinks per drinking occasion and three or more days per week was more common among SB cases, this pattern was due to confounding by FA intake, study center and period, and education. With regard to the impact of heavy drinking during the first 28 days after LMP, we found no association with the risk of SB. Results of previous studies using similar categories of exposure have been inconclusive. One study observed an increase in the risk of NTDs (OR: 1.7, 95% CI: 0.8, 3.6) [11] while two studies of SB reported null results [14,17].

### 4.3. Coffee

We found that maternal exposure to caffeine from coffee was not associated with an increased risk of SB. Rather, ORs for varying levels of intake were all below 1.0, but the 95% confidence intervals included 1.0. Furthermore, we observed very little confounding of our risk estimates for caffeine. Consistent with our findings, two previous studies found no association between NTDs and coffee or caffeine [13,35] and one found a suggestion of a protective effect [36]. In one previous study, there was a slight increase in the risk for SB among mothers who consumed coffee [12]. However, similar to our finding, there was no dose effect observed, which the authors cited as evidence against a causal association [12].

### 4.4. Smoking, Alcohol, and Coffee Interaction

The combination of smoking 10+ cigarettes per day and heavy drinking was not associated with SB risk in the earlier years of the study, but a doubling in risk in the later years. As described above in our discussion of cigarette smoking results, the discontinuity in our findings between the two time periods could be due to different interviewing formats or uncontrolled confounding. Previous studies of smoking and alcohol exposures in relation to NTDs did not examine the interaction of these two variables [14,17,42,43], but one study did show an increase (aOR: 12.7 95% CI: 3.5, 45.3) for the risk of congenital cardiac defects and the interaction of maternal binge drinking (≥5 drinks on at least one occasion) and smoking (yes, no) in the three months prior to pregnancy [47].

### 4.5. Bias/Limitations

There were limitations to our study. First, reported exposures suggest that only a small percentage of women are in the highest exposure group, which limits our ability to study the effects of these exposures and decreases the precision of our estimates. The problem was exacerbated when the data were stratified by FA intake. Additionally, data on exposure were collected by maternal self-report which likely has high sensitivity but low specificity in that some women may under-report. Such misclassification may be greater for exposures with the most stigma [48] in pregnancy (e.g., cigarettes and alcohol). If mothers of cases are more likely to deny high level use, effect estimates would be biased downward. Studies have shown that reliability of self-reported smoking during pregnancy is generally good [49,50,51], but subjects may underreport the amount they smoke [52,53]. Alcohol intake in pregnancy was found to be under-reported by 44% in one study [48]. In a study examining coffee intake reporting, it was found that amounts reported by FFQ correlated well with amounts reported by daily food diaries; however, absolute intake differed between the FFQ and the daily food diary [54]. Accuracy of reporting exposure to cigarettes, alcohol, and coffee is further complicated by our early pregnancy exposure window, which is a time when women often change their exposure patterns. Further, exposures in the earliest days of lunar month two may be etiologically relevant but were not considered in our exposure algorithms. Therefore, we cannot rule out the possibility of misclassification of our exposures.

Power was maximized and bias minimized through systematic approaches in the study design. This study included a large number of cases and controls to maximize the number of subjects within each stratum of exposure. During maternal interviews, information was gathered on frequency, quantity, and timing through the use of a standardized questionnaire and detailed support material (e.g., calendars) to help participants give complete and accurate responses. To minimize any potential bias stemming from maternal recall, reporting accuracy was maximized for both cases and controls through highly structured interviews conducted within six months of birth or termination by skilled and experienced nurse interviewers who were unaware of the study hypotheses.

## 5. Conclusions

We have investigated the association between the risk of SB and periconceptional cigarette, alcohol, and coffee consumption, using a large geographically diverse case-control study. Our findings suggest that there is no increased risk for SB among women who consumed cigarettes, alcohol, and caffeine. This observation held true among women who did not consume the recommended amount of folic acid. Despite the fact that our findings are similar to those of previous studies, the results should still be interpreted cautiously due to limitations, including low precision for the highest levels of intake.
